# Characteristics of Rod Regeneration in a Novel Zebrafish Retinal Degeneration Model Using N-Methyl-N-Nitrosourea (MNU)

**DOI:** 10.1371/journal.pone.0071064

**Published:** 2013-08-12

**Authors:** Christoph Tappeiner, Jasmin Balmer, Matias Iglicki, Kaspar Schuerch, Anna Jazwinska, Volker Enzmann, Markus Tschopp

**Affiliations:** 1 Department of Ophthalmology, Inselspital, University of Bern, Bern, Switzerland; 2 Department of Ophthalmology, Hospital de Clinicas, University of Buenos Aires, Buenos Aires, Argentina; 3 Department of Biology, University of Fribourg, Fribourg, Switzerland; 4 Department of Ophthalmology, University Hospital of Basel, Basel, Switzerland; Wayne State University School of Medicine, United States of America

## Abstract

Primary loss of photoreceptors caused by diseases such as retinitis pigmentosa is one of the main causes of blindness worldwide. To study such diseases, rodent models of N-methyl-N-nitrosourea (MNU)-induced retinal degeneration are widely used. As zebrafish (Danio rerio) are a popular model system for visual research that offers persistent retinal neurogenesis throughout the lifetime and retinal regeneration after severe damage, we have established a novel MNU-induced model in this species. Histology with staining for apoptosis (TUNEL), proliferation (PCNA), activated Müller glial cells (GFAP), rods (rhodopsin) and cones (zpr-1) were performed. A characteristic sequence of retinal changes was found. First, apoptosis of rod photoreceptors occurred 3 days after MNU treatment and resulted in a loss of rod cells. Consequently, proliferation started in the inner nuclear layer (INL) with a maximum at day 8, whereas in the outer nuclear layer (ONL) a maximum was observed at day 15. The proliferation in the ONL persisted to the end of the follow-up (3 months), interestingly, without ongoing rod cell death. We demonstrate that rod degeneration is a sufficient trigger for the induction of Müller glial cell activation, even if only a minimal number of rod cells undergo cell death. In conclusion, the use of MNU is a simple and feasible model for rod photoreceptor degeneration in the zebrafish that offers new insights into rod regeneration.

## Introduction

Zebrafish (Danio rerio) provide an important model system in visual research, especially due to their cone-rich retina and persistent retinal neurogenesis throughout the zebrafish lifetime [1]–[6]. Under physiological conditions, the ciliary marginal zone (CMZ) is the source of all types of neurons in the adult zebrafish retina [7]. In addition, Müller glial cells located in the inner nuclear layer (INL) of the retina are able to generate rod progenitors, which reside in the outer nuclear layer (ONL) [7]. These two sources provide lifelong retinal growth and neurogenesis, which is necessary to maintain a stable rod density in a continuously growing eye [8]–[12]. When retinal damage occurs, cells in the INL give rise to multipotent progenitors, which proliferate and substitute all types of neurons to reconstitute the previous tissue architecture [7], [11], [13]–[17]. Barnados et al. have investigated these proliferating cells in an ultra-high-intensity light treatment model in transgenic zebrafish in which Müller glial cells expressed green fluorescence protein [18], [19]. They concluded that the proliferating cells in the INL were de-differentiated Müller glial cells and are able to migrate from the INL into the ONL forming rod progenitors and thereby regenerating photoreceptor cells [7], [18], [19]. In a constant-light damage model, approximately 50% of Müller glial cells co-labeled for PCNA, a marker for mitosis [7], [15]. Injection and in vivo electroporation of PCNA morpholinos inhibited Müller glial cell proliferation and blocked regeneration of the retina. These data suggest that Müller glial cell division is necessary for proper photoreceptor regeneration in the light-damaged zebrafish retina and that Müller glial cells serve as the source of neuronal progenitor cells [15]. The difference of these two light-induced models (ultra-high-intensity vs. constant-light induced damage model) were analysed by Thomas et al. [20]. Whereas constant bright light primarily damages rod photoreceptors, ultra-high treatment with UV light results in significant damage to both rods and cones [20]. In mechanically induced injury to the retina (either with a needle [21] or by surgically removing a small part of the retina [22]), the same proliferating Müller glial cells were observed replacing the damaged retina. Müller glial cells also have the potential to regenerate neurons of the inner retina, as observed in a model of inner retina destruction with intravitreal injection of ouabain [14] or after optic nerve crush [17].

In rodents, retinotoxic substances such as N-methyl-N-nitrosourea (MNU) are used to induce photoreceptor degeneration [23], [24]. MNU is an alkylating agent, widely used as an experimental cancer-inducing drug leading to benign and malignant tumors, that appear several months after MNU treatment [25]–[27]. In addition, MNU specifically induces photoreceptor damage in rodents shortly after exposure [23], [24], presumably directly caused by the alkylating DNA damage of the outer nuclear layer [28]. However, it remains a puzzling question why photoreceptors, especially rods, are particularly susceptible to MNU [23], [24]. A possible explanation may be the very low concentration (or absence) of glutathione in photoreceptors [29]. Glutathione both catalyzes the decomposition of MNU and scavenges the produced methylating agent [29], [30].

In a new approach, we examined the use of MNU in adult zebrafish in order to establish a simple chemically induced model for photoreceptor degeneration.

## Materials and Methods

### Animals

Wild-type zebrafish (Danio rerio) of the AB (Oregon) strain aged 12 to 24 months were used. The fish were maintained in standard conditions [31], [32] in water with a temperature of approximately 26.5° Celsius and raised in a 14/10 hour light/dark cycle. The experimental research on animals was approved by the Cantonal Veterinary Office in Fribourg and adhered to the Association for Research in Vision and Ophthalmology (ARVO) Statement for the Use of Animals in Ophthalmic and Vision Research.

### MNU treatment protocol

At day 0, zebrafish were incubated in fresh water containing 10 mM phosphate buffer, pH 6.3, with a concentration of 50 mg/l (group 1) or 150 mg/l (group 2) dry substance of MNU (Sigma, St. Louis, USA) for 60 minutes. Following exposure, fish were washed and thereafter maintained under standard conditions.

### Histology and cell quantification

After euthanasia, the eyes were enucleated before and at day 1, 3, 5, 8, 15, 30, 60 or 90 after MNU treatment and fixed for at least 18 hours with 4% paraformaldehyde in phosphate-buffered saline (PBS). Then, the eyes were embedded in paraffin, and 5 μm sections were cut. Sagittally oriented central sections of the optic nerve head were used for measurement purposes. The sections were stained with hematoxylin and eosin (H&E). In the right eyes of all of the fish, the number of cells in the ganglion cell layer and the inner nuclear layer (INL) as well as the number of rods and cones were determined manually at the same position in the midperiphery on both sides of the eye (size of the counted area refers to a retinal section of 150 μm length). Rod and cone photoreceptors were distinguished by their different morphologies and position [33], [34].

### TUNEL and immunohistochemistry

Paraffin tissue sections were also used for TUNEL staining and immunohistochemistry. An in situ cell death detection kit (TMR red, Roche Applied Science, Rotkreuz, Switzerland) was applied to detect TUNEL-positive cells within the retina (n = 3 eyes of 3 zebrafish for all time points). When immunohistochemistry was combined with TUNEL staining, immunohistochemistry was performed first (as described below; dilution of anti-rhodopsin antibody 1∶200, dilution of anti-zpr-1 antibody 1∶200) and directly followed by TUNEL staining.

Immunohistochemistry was performed with the following primary antibodies: rabbit anti-PCNA (to detect cell proliferation; 1∶200 dilution; Santa Cruz Biotechnology, Santa Cruz, USA), rabbit anti-GFAP (to detect activated Müller glial cells; 1∶200; Life Technologies, Paisley, UK), mouse anti-rhodopsin (to detect rod photoreceptors; 1∶200; NeoMarkers, Fremont, USA) or mouse anti-zpr-1 (to detect red-green double cones, 1∶50; Zebrafish International Resource Center, Oregon, USA) in Tris-buffered saline (TBS) with 1% bovine serum albumin (BSA) for 1 hour at room temperature or overnight at 4° Celsius. As secondary antibodies, goat anti-rabbit/anti-mouse Alexa 488 nm/594 nm (1∶500; Life Technologies, Paisley, UK) was applied in TBS with 1% BSA for 1 hour at room temperature. To identify proliferating cells, histological sections were double-stained with the above-mentioned antibodies for PCNA in addition to GFAP, rhodopsin and zpr-1, respectively. The first sequence was blocked with TBS and 10% goat normal serum and 1% BSA, and the second sequence was blocked with TBS, 5% mouse normal serum and 1% BSA.

Cell proliferation was assessed in all eyes by counting PCNA-positive cells according to the method used for counting cells using H&E staining (size of the counted area refers to a retinal section of 450 μm length). In addition, the number of PCNA-positive cells in the CMZ, which was defined as the unlaminated retina, was counted.

### Statistical analysis

Statistical analysis was performed using GraphPad software (version 5.0 c, GraphPad Software, La Jolla, USA). Intergroup comparisons were based on a 1-way analysis of variance (ANOVA) and the Bonferroni multiple comparison post hoc test. The level for statistical significance was set at a P value of 0.05. Cell counts were performed in 6 eyes of 6 zebrafish for the control group and in 3 eyes of 3 zebrafish for all other time points. For each eye, cells of two corresponding areas (opposite sides of the optic nerve) were counted and mean values were calculated.

## Results

**Figure 1:**
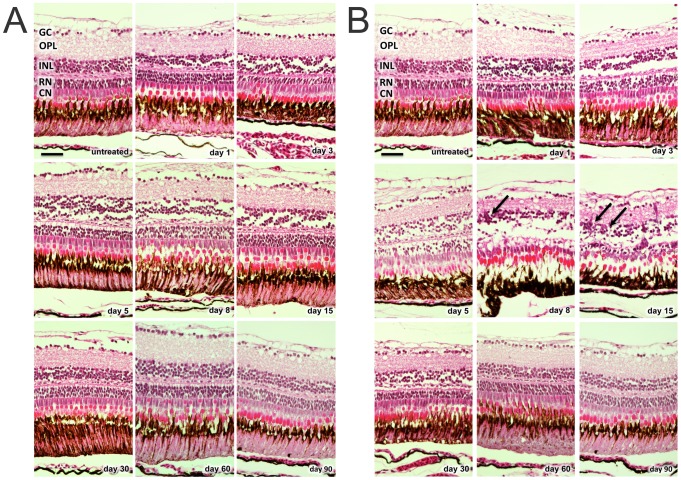
H&E staining of zebrafish retinas at baseline and after MNU exposure. A. MNU 50 mg/l group: only minor changes of retinal structure were observed after MNU treatment (e.g., disruption of the inner nuclear layer and vacuolation of the outer nuclear layer between day 1 and 30). B. MNU 150 mg/l group: Histological changes already started at day 1 (e.g., disruption of the inner nuclear layer and vacuolation of the outer nuclear layer), resulting in massive retinal degeneration with loss of nearly all rod cells at day 8. On days 8 and 15, accumulations of cell clusters (arrows) were found mainly in the inner nuclear layer. GC (ganglion cells), OPL (outer plexiform layer), INL (inner nuclear layer), RN (rod nuclei), CN (cone nuclei). Scale bar indicates 25 μm.

**Figure 2:**
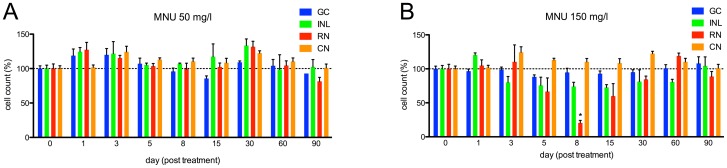
Cell counts in different retinal layers for zebrafish exposed to MNU at baseline and follow-up. A. MNU 50 mg/l group: No relevant decrease in retinal cells was observed (p>0.05). B. MNU 150 mg/l group: Rod cell loss started at day 5; the number of rods was lowest at day 8 (p≤0.01) with a decrease of 79.6%, but fully recovered by day 60. Other retinal layers did not display any relevant decrease of cell numbers after MNU exposure (p>0.05). GC (ganglion cells), INL (inner nuclear layer), RN (rod nuclei) and CN (cone nuclei). Baseline values are defined as 100%. Mean values with SEM error bars are represented (* indicates p≤0.01 compared with day 0).

In the 50 mg/l group, only minor changes of retinal structure were observed after MNU treatment (e.g., disruption of the INL and vacuolation of the ONL between day 1 and 30; **Fig. 1A**). Furthermore, no relevant cell count decline of any retinal layer occurred during follow-up (p>0.05; **Fig. 2A**). H&E staining in the 150 mg/l group revealed histological changes starting at day 1 (e.g., disruption of the INL and vacuolation of the ONL), resulting in massive retinal degeneration that involved a loss of nearly all rod cells at day 8 (reduction of 79.6% compared to control retina, p≤0.01; **Fig. 1B** **, and** **2B**). Although all retinal layers displayed morphological changes after MNU treatment, only rods displayed distinct cell loss. Significant decreases were not observed for the other retinal layers (p>0.05). However, cells in the INL showed a non-significant reduction between day 3 and 60. On days 8 and 15, accumulations of cell clusters were found, mainly in the INL (**Fig. 1B** **, arrows**). These cells stained for proliferating cell nuclear antigen (PCNA), which is a proliferation marker (see below).

**Figure 3:**
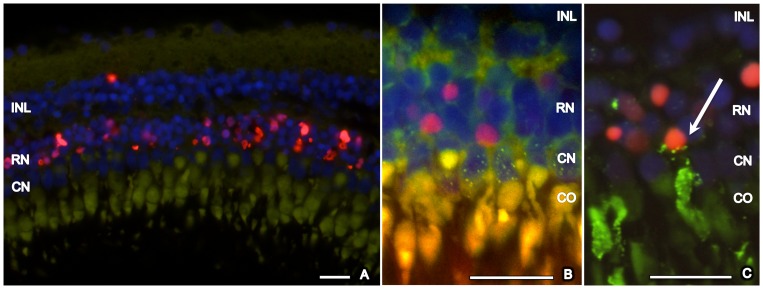
TUNEL staining of zebrafish retina, 3 days after exposure to MNU 150 mg/l. A. TUNEL-positive cells (red) are localized in the outer nuclear layer. Strong autofluorescence (green) of a cone outer segment allows identification of the corresponding nearby cone nucleus. Based on this assessment, cone photoreceptors are TUNEL-negative. B. Immunhistochemistry with zpr-1 (staining double cones, green dotted) combined with TUNEL staining (red) confirmed that cone photoreceptors are TUNEL-negative. C. Immunhistochemistry with rhodopsin (staining rods, green) and TUNEL staining (red). Co-Localization of rhodopsin and TUNEL exemplarily shows a dying rod photoreceptor (arrow). Cell nuclei are stained with DAPI (blue). INL (inner nuclear layer), RN (rod nuclei), CN (cone nuclei), CO (cone outer segment). Scale bar indicates 50 μm.

Following treatment with 50 mg/l MNU, no TUNEL-positive cells were seen at baseline or any other time point. In contrast, zebrafish exposed to 150 mg/l displayed many TUNEL-positive cells on day 3 that were located mainly in the ONL (**Fig. 3A**). Maximal one (mainly no) TUNEL-positive cell per histological section was found at any other time point. Strong autofluorescence of a cone outer segment allows identification of the corresponding nearby cone nucleus. Based on this assessment, cone photoreceptors were TUNEL-negative. To verify this, immunohistochemistry with zpr-1 (staining double cones) combined with TUNEL staining was performed (**Fig. 3B**). Again, red-green double cones were TUNEL-negative. We also assessed directly if TUNEL-positive cells are rod photoreceptors by performing a combined staining with rhodopsin. Although rhodopsin mainly stains the outer segments of rod photoreceptors (in contrast to the labeling of apoptotic nuclei by TUNEL), co-localization was observed for some rod photoreceptors, especially when the outer segment was displaced close to the nucleus (**Fig. 3C**). Taking all together, it can be concluded that TUNEL-positive cells localized in the ONL are rod photoreceptors.

**Figure 4:**
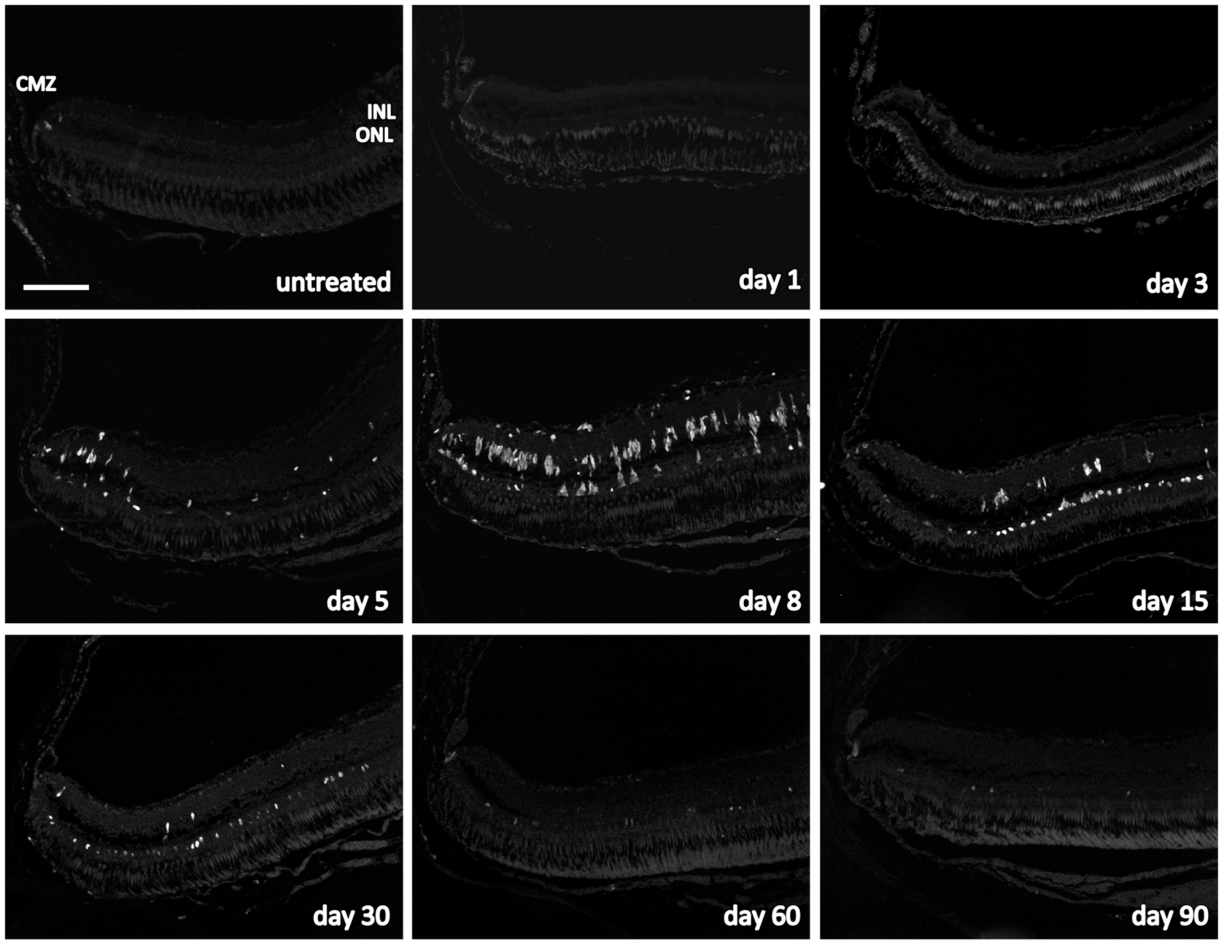
Cell proliferation in the zebrafish retina exposed to 150 mg/l MNU. Proliferating cell nuclear antigen (PCNA) positive cells (white) indicate proliferation and were found in the ciliary marginal zone (CMZ) at all time points and in the untreated fish. Furthermore, PCNA-positive cells were observed in the inner nuclear layer (INL) starting at day 3 and their number was highest at day 8. Proliferating cells in the outer nuclear layer (ONL) were not found before day 5 but their number was highest at day 15. From day 30 on, nearly no proliferating cells were seen in the INL, whereas proliferation in the ONL occurred until the end of follow up at day 90. Scale bar indicates 100 μm.

**Figure 5:**
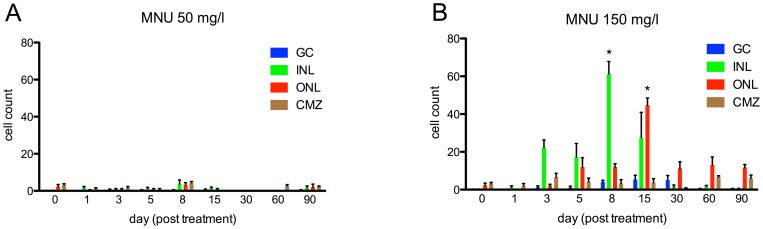
Quantification of cell proliferation in zebrafish retina exposed to MNU. A. Before MNU exposure (day 0) few PCNA-positive cells were found in the ciliary marginal zone (CMZ) and the outer nuclear layer (ONL). Additionally, in the MNU 50 mg/l group, few PCNA-positive cells were observed in the inner nuclear layer (INL) at day 8, whereas at all other time points, no relevant signs of proliferation were found. Ganglion cells (GC) revealed no relevant cell proliferation. B. In the 150 mg/l group, PCNA-positive cells were seen in the INL starting at day 3, and their number was highest at day 8 (p≤0.0001). Proliferating cells in the ONL were not found before day 5, but their number was highest at day 15 (p≤0.0001). From day 30 on, almost no proliferating cells were observed in the INL, whereas proliferation in the ONL occurred until the end of the follow up at day 90 Cell counts refer to retinal sections of 450 μm length. Mean values with SEM error bars are represented (* indicates p≤0.0001 compared with day 0).

**Figure 6:**
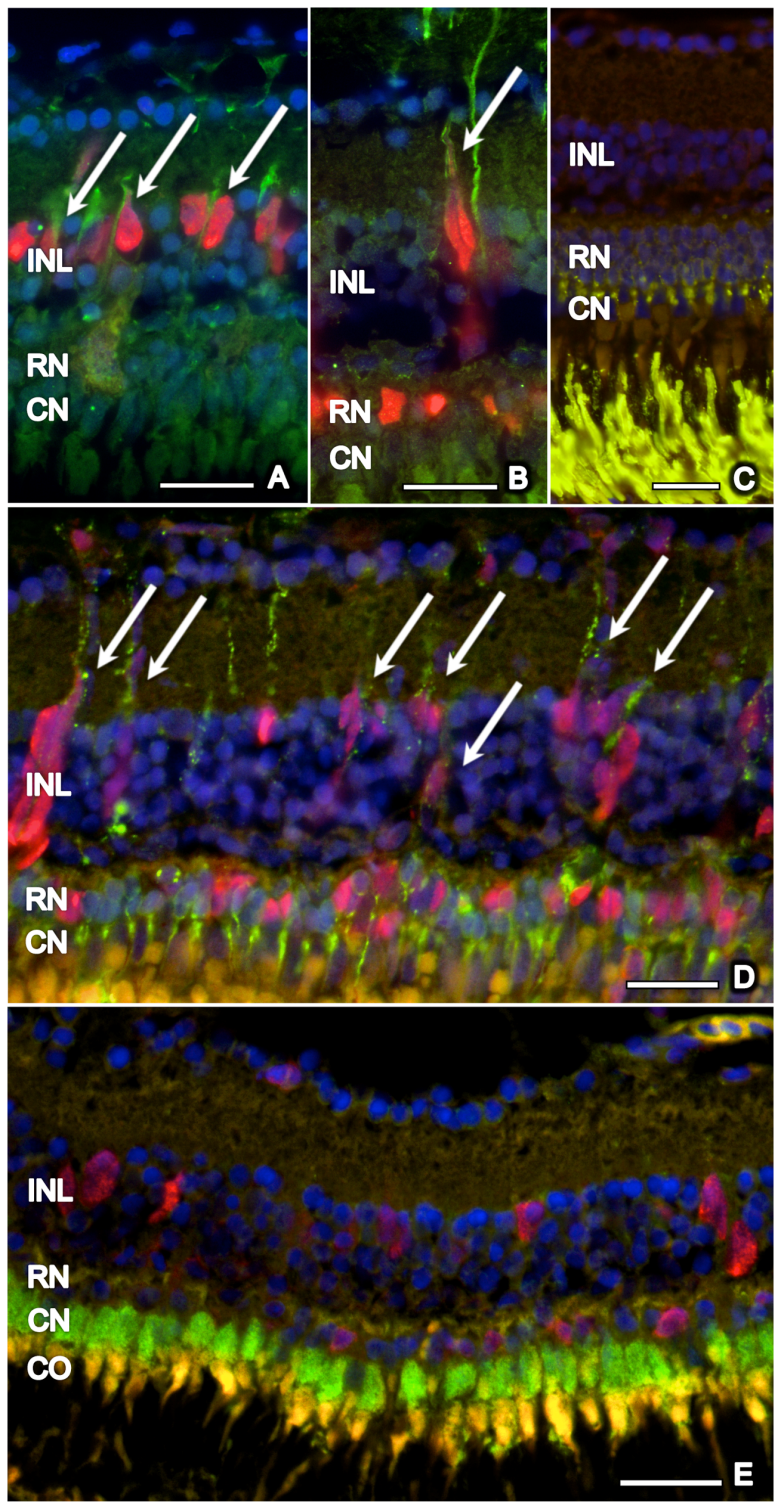
Co-localization of proliferating cells with GFAP, rhodopsin, and zpr-1. Proliferating (PCNA-positive, red) cells in the inner nuclear layer (INL), but not in the outer nuclear layer (ONL: rod nucleus  =  RN, cone nucleus  =  CN), co-localized with GFAP (green) (arrows) at days 5 (A) and 15 (B). No rhodopsin is observed in the INL of untreated retina (C), whereas many PCNA-positive cells co-localized (arrows) with rhodopsin (green) in the ONL and INL during retinal regeneration after MNU exposure (D; day 15). No co-localization of zpr-1 stained double cones (green) and PCNA (red) was found (E, day 5). Cell nuclei are stained with DAPI (blue). Scale bar indicates 50 μm.

Before MNU exposure, few PCNA-positive cells were found in the ciliary marginal zone and the ONL (**Fig. 4** **and** **5**). In addition, the INL showed few PCNA-positive cells in the 50 mg/l group at day 8 (**Fig. 5A**). In the 150 mg/l group, proliferating PCNA-positive cells were detected in the INL starting at day 3 and reached a maximum at day 8 (p≤0.0001; **Fig. 4** **and** **5B**). PCNA-positive cells in the ONL were not found before day 5 but reached a maximum at day 15 (p≤0.0001). From day 30 on, almost no PCNA-positive cells were observed in the INL, whereas proliferation in the ONL occurred until the end of the follow up at day 90 (**Fig. 4** **and** **5B**). About 40–70% (depending on the histological section; assessed for day 5 to 15) of the PCNA-positive cells in the INL (but none in the ONL) were co-localized with glial fibrillary acidic protein (GFAP), which is a marker for glial cells including activated Müller glial cells (**Fig. 6A and 6B**). Accordingly, the shape of these proliferating cells in the INL was similar to that of Müller glial cells. Whereas no rhodopsin was found in the INL of the untreated control retina (**Fig. 6C**), about 30–50% of dividing cells in the INL showed rhodopsin staining (**Fig. 6D**). At all time points, PCNA-positive cells in the INL and ONL were not co-localized with zpr-1, which stains red-green double cones (**Fig. 6E**; data shown for day 5).

## Discussion

Primary photoreceptor loss is one of the major causes of visual loss, and retinitis pigmentosa is a typical example of such a retinal degenerative disease [35], [36]. To identify the underlying pathomechanisms, different animal models have been developed. One of the established pharmacological models is the induction of photoreceptor death by MNU in rodents [23], [24]. Here, we introduce the use of MNU to study retinal degeneration and subsequent regeneration in the adult zebrafish, which is an increasingly popular model because its ease of maintenance, high fecundity and, most notably, cone-rich retina. In contrast with mammals, zebrafish undergo persistent retinal neurogenesis throughout their lifetimes and also retinal regeneration after severe damage [22], [37]–[40]. Analyzing the mechanisms of retinal regeneration in the zebrafish may identify cells and pathways with the potential for regeneration, and allows to study differences between zebrafish and other species without such regeneration. With this in mind, we expanded the MNU model to adult zebrafish. To the best of our knowledge, this is the first chemical model in zebrafish that induces only photoreceptor degeneration. In contrast, existing models (e.g., ouabain) lead to damage of the inner or whole retina [14], [40], [41]. The MNU model is less invasive than existing models that employ intravitreal injection of substances, such as the ouabain model, or surgically induced techniques [14], [22]. MNU can be dissolved directly in the tank water of the zebrafish without the need for intraperitoneal or intravitreal injections, which may also lead to better reproducibility. In contrast to light-induced retinal degeneration models [20], our model is more specific to rods without any significant cone loss. The comparison of these models may help to decipher the specific molecular pathways that are involved in rod versus cone photoreceptor regeneration.

In this study, we demonstrated a characteristic sequence of retinal changes. First, histology revealed apoptosis of rod photoreceptors 3 days after MNU treatment that resulted in reduced rod cell counts starting at day 5 that reached a minimum at day 8. Subsequently, proliferation started in the INL at day 3 and reached a maximum at day 8. In the ONL, the first proliferating cells were observed at day 5 and reached a maximum at day 15. Thereafter, increased proliferation persisted in the ONL up to the end of the study, whereas increased proliferation in the INL was not observed after day 15. Considering the earlier peak of proliferation in the INL compared to the ONL and rhodopsin expression in proliferating cells of the INL, it may be presumed that proliferating (Müller glial) cells migrate from the INL to the ONL. This is in accordance with previous studies [7], [18], [19], [42], [43]. We assume that also rod progenitors may contribute to the regeneration of the retina and are the main source of proliferation at later time points, since no further PCNA-positive cells can be observed in the INL after day 15.

In previous studies, regenerating cells in the INL were reported to be Müller glial cells [44], [45]. Similarly, in our study, dividing cells in the INL displayed a Müller glial cell like morphology and were co-localized with GFAP, which is a marker for glial cells including activated Müller glial cells. Dividing cells in the ONL did not co-localize with GFAP or zpr-1 (staining red-green double cones), which agrees with the findings that cones were not TUNEL-positive and that H&E staining did not reveal a loss of cones. In contrast, rod cells were TUNEL-positive and demonstrated dramatic cell loss (about 80% at day 8). During the subsequent rod regeneration, dividing cells were co-localized with rhodopsin. Interestingly, in addition to cells in the ONL, also several dividing cells in the INL expressed rhodopsin. This may indicate that their fate to differentiate into rod photoreceptors is already determined in the INL. Alternatively, the rhodopsin staining in the INL may be caused by Müller glial cells that clear dying rod photoreceptors. Intriguingly, in a mice model of inherited retinal degeneration, Müller glial cells also express rhodopsin [46]. Taken all together, our findings confirm that Müller glial cells have the potential to differentiate into rod photoreceptors, at least in zebrafish. Recently, similar findings were also obtained in vitro with human Müller glial cells [47].

In summary, our MNU model in the zebrafish demonstrates loss and regeneration of rods. Other cell types, including cones, were not affected. This agrees with the results of Boudard et al., who showed that rods are more susceptible to MNU damage than cones in the diurnal cone-rich rodent Arvicanthis ansorgei [48], [49]. To the best of our knowledge, there is no other chemical model that induces selective rod degeneration in the zebrafish.

Previously, a model of rod degeneration in the Tg(Xops:mCFP) transgenic zebrafish line suggested that rod degeneration alone is not able to induce Müller glial cell proliferation [50]. In contrast, Montgomery et al. presented two models of rod degeneration in transgenic zebrafish in which rod degeneration is activated by metronidaziole: the Tg(zop:nfsB-EGFP)^nt19^ zebrafish showed acute loss of all rods followed by Müller glial cell activation, whereas in the Tg(zop:nfsB-EGFP)^nt20^ model, only a subset of rods was ablated, which resulted in activation of rod progenitors, and not Müller glial cells [51]. Montgomery et al. postulated that the extent of rod cell death regulates the origin of regenerated rod photoreceptors and noted the following prerequisites for Müller glial cell activation [51]: massive rod cell death, acute rod loss and/or loss of fully differentiated rods. Our data confirm that rod cell loss is sufficient to induce Müller glial cell activation. We have further been able to demonstrate that even the death of only few rod photoreceptors triggers Müller glial cell activation. The acute death of fully differentiated rods in our model, as opposed to chronic loss of premature rod photoreceptors observed in Tg(zop:nfsB-EGFP)^nt20^ zebrafish, may explain this difference.

In our model, PCNA-positive cells were observed in the ONL (but not in the INL) up to 3 months after MNU damage. Although other reasons cannot be excluded, it is highly suggestive that the observed PCNA-positive cells are proliferating rod progenitors. Remarkably, no further ongoing detectable rod cell death was observed (which is known to induce proliferation of rod progenitors [13]). The reason for this prolonged proliferation is unknown; it might be that low rod cell density alone may trigger rod progenitors activation [12], but not Müller glial cell activation.

In the rodent Arvicanthis ansorgei, Boudard et al. revealed a secondary loss of cones three months after MNU treatment in addition to the early death of rod photoreceptors immediately after MNU treatment [48]. This secondary cone loss has been observed in many species with different degeneration models and in retinal degenerative diseases in humans [52]–[55]. Whether this secondary cone loss is caused by deprivation of essential neurotrophic factors or results from “poisoning” by the death of rod photoreceptors is an ongoing debate [50], [56], [57]. The present model showed massive rod photoreceptor death. However, there was no secondary death of cone photoreceptors. We speculate that the absence of the secondary loss of cones in our model may have been the result of rapid and efficient rod regeneration. This speculation supports the hypothesis that the secondary loss of cones in other models of retinal degeneration is caused by deprivation of essential neurotrophic factors arising from a prolonged absence of rods and not by the “poisoning” of cone photoreceptors.

In conclusion, we present a simple and feasible model for inducing rod photoreceptor degeneration in zebrafish; this model is comparable to degeneration previously described in rodents. Why subsequent regeneration occurs only in certain species and not in others remains a puzzling and fascinating question.
